# Supplementary material for: Interactive Effects of Flooding Duration and Sediment Texture on the Growth and Adaptation of Three Plant Species in the Poyang Lake Wetland
Source: Biology (Basel). 2023 Jul 1;12(7):944. doi: 10.3390/biology12070944 (PMC10376433; doi:10.3390/biology12070944)
Supplement: Supplementary file 1 [file biology-12-00944-s001.zip › biology-2377715-supplementary.pdf]

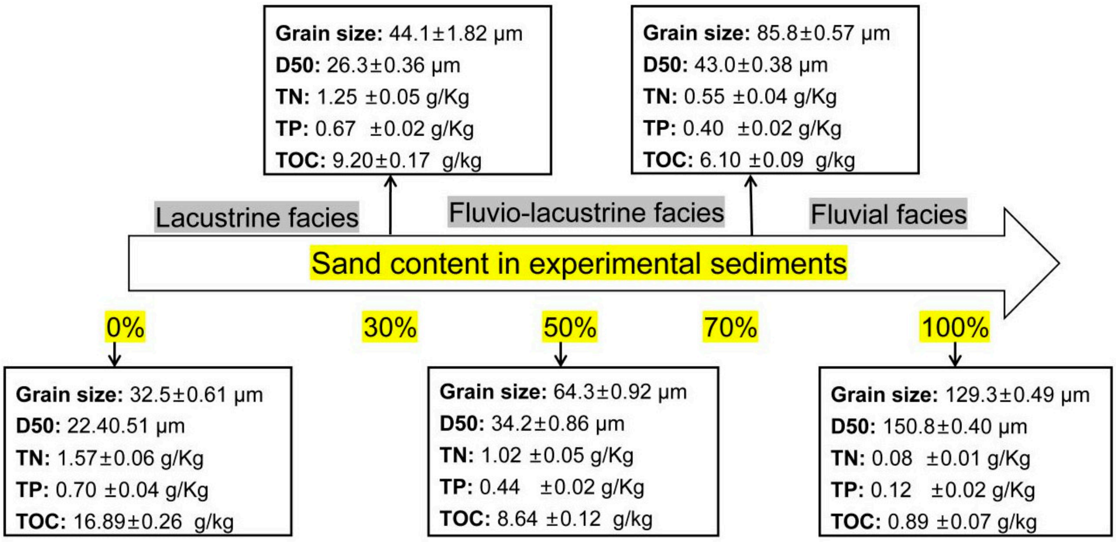

Figure S1. Physicochemical properties of five sediments with differing texture. The five sediments could simulate the conditions of different soil sediment types distributed in the shoals and beaches in Poyang Lake region. D50: median particle size; TN: total nitrogen content of sediments; TP: total phosphorus content of sediments; TOC: total organic carbon content of sediment.
